# Heterologous Omicron-adapted vaccine as a secondary booster promotes neutralizing antibodies against Omicron and its sub-lineages in mice

**DOI:** 10.1080/22221751.2022.2143283

**Published:** 2022-12-12

**Authors:** Jianyang Liu, Qian He, Fan Gao, Lianlian Bian, Qian Wang, Chaoqiang An, Lifang Song, Jialu Zhang, Dong Liu, Ziyang Song, Lu Li, Yu Bai, Zhongfang Wang, Zhenglun Liang, Qunying Mao, Miao Xu

**Affiliations:** a National Institutes for Food and Drug Control, Beijing, People’s Republic of China; b Guangzhou Laboratory, Guangzhou, People’s Republic of China

**Keywords:** COVID-19, Omicron variant, booster vaccine, neutralizing antibody, heterologous vaccination

## Abstract

Over one billion people have received 2–3 dosages of an inactivated COVID-19 vaccine for basic immunization. Whether a booster dose should be delivered to protect against the Omicron variant and its sub-lineages, remains controversial. Here, we tested different vaccine platforms targeting the ancestral or Omicron strain as a secondary booster of the ancestral inactivated vaccine in mice. We found that the Omicron-adapted inactivated viral vaccine promoted a neutralizing antibody response against Omicron in mice. Furthermore, heterologous immunization with COVID-19 vaccines based on different platforms remarkably elevated the levels of cross- neutralizing antibody against Omicron and its sub-lineages. Omicron-adapted vaccines based on heterologous platforms should be prioritized in future vaccination strategies to control COVID-19.

## Main

Novel coronavirus disease 2019 (COVID-19) is caused by severe acute respiratory syndrome coronavirus 2 (SARS-CoV-2), which is primarily transmitted via aerosols [1]. COVID-19 has led to over 608 million confirmed cases and at least 6.5 million deaths worldwide [2]. Vaccination is considered an effective strategy for preventing COVID-19. However, the Omicron variant carries over 30 mutations in the spike protein, and can easily escape from vaccine-induced SARS-CoV-2 neutralizing antibodies[3]. Strategies such as homologous prime-boost, heterologous prime-boost, and Omicron variant-adapted vaccine development have been suggested for preventing symptomatic Omicron infections [4,5]. Over one billion people have been injected with 2–3 dosages of inactivated virus vaccines [6]. However, whether a booster immunization strategy should be adopted for Omicron and its sub-lineages remains unclear. Manufacturers have initiated pre-clinical and clinical studies of Omicron-adapted vaccines based on various technology platforms, such as inactivated virus and mRNA vaccines [7,8]. Here, we examined the immunogenicity of different Omicron-adapted COVID-19 vaccines as a secondary booster for basic immunization with wild-type inactivated virus vaccines (IV-WT).

**Figure 1.**
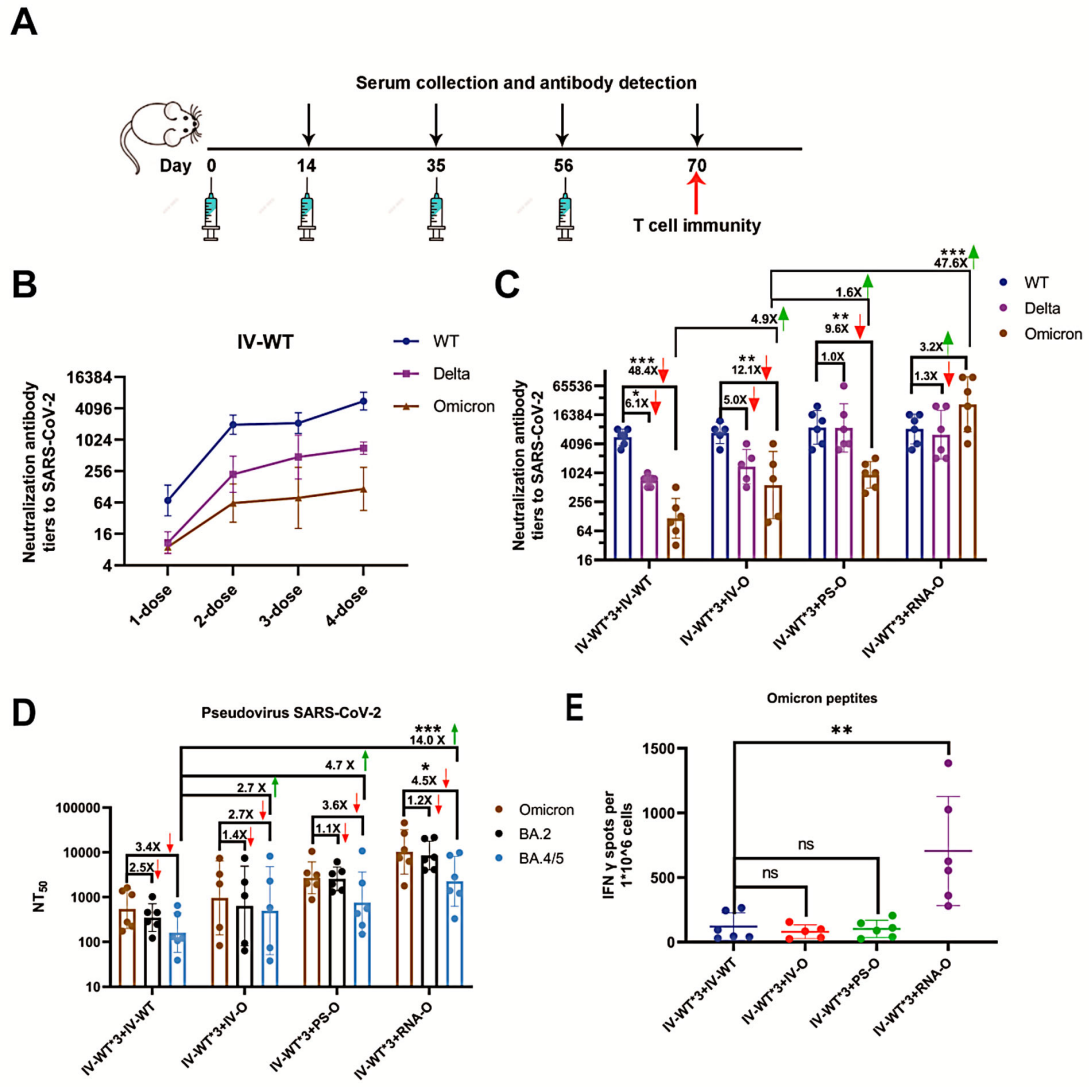
Booster doses of Omicron-adapted vaccines enhance the Nab response in mice. (A) Scheme of immunizations, serum collection, and T cell draws. (B) Nabs titers to WT virus at days 14, 35, 56, and 70. (C) Nabs titers to WT, Delta, and Omicron viruses at day 70. (D) Serum Nabs titers against pseudoviruses displaying Omicron spike proteins, BA.2, and BA.4/5 at day 70. (E) ELISPOT assay for IFN-γ in splenocytes. The red downward arrows represent a decreasing fold change and the green upward arrows represent an increasing fold change. (B–D) Bars represent geometric means, and error bars represent geometric standard deviations for each group. Statistical comparisons across groups were determined by one-way ANOVA with Bonferroni’s multiple comparisons test after log transformation. (E) Bars represent means ± SD. Statistical comparisons across groups were determined by one-way ANOVA with Tukey’s multiple comparisons test. (*p < 0.05; **p < 0.01; ***p < 0.001).

We designed a four-dose vaccination schedule to evaluate the efficacy of the booster dose (Figure 1A). A homologous booster dose with IV-WT induced high levels of cross- neutralizing antibodies (Nabs) (Figure 1B). However, a secondary booster dose with an IV-WT vaccine had minimal additional effects, and a neutralizing geometric mean titer (GMT) against Omicron of 116(95%CI: 43-315), 48- and 6.1-fold lower than those against the prototype and Delta variant, respectively. These results indicate that a second booster dose with IV-WT is ineffective against symptomatic infections of Omicron.

As an alternative to a second dose of IV-WT, three Omicron-adapted COVID-19 candidate vaccines based on various platforms, namely, inactivated-Omicron vaccine (IV-O), protein subunit-Omicron vaccine (PS-O), and mRNA-Omicron vaccine (RNA-O), were used for vaccination individually following three IV-WT vaccines. The neutralizing GMTs against the WT were not significantly different among the four vaccination schedules, but were increased against Delta and Omicron for the three Omicron-adapted candidate vaccines compared to the IV-WT. Mice immunized with three doses of IV-WT followed by immunization with the IV-O vaccine showed a neutralizing GMT of 568(95%CI: 77-4197) for Omicron, which was 4.9-fold higher than after the IV-WT vaccine (Figure 1C). Remarkably, heterologous vaccination with three doses of IV-WT followed by PS-O and RNA-O boosters had neutralizing GMTs of 930(95%CI: 476-1817) and 27,049(95%CI: 6784-107875) for Omicron, respectively. Compared to IV-O, PS-O and RNA-O induced 1.6- and 47.6-fold increases in neutralizing activity against Omicron (Figure 1C). These results demonstrate that a second booster vaccination with a heterologous Omicron-adapted vaccine can elicit potent broad neutralizing activity against SARS-CoV-2.

We performed a pseudovirus neutralization assay to assess Nabs resistance to Omicron sub-lineages. The neutralizing GMTs against BA.4/5 were 161(95%CI: 55-470), 440(95%CI: 52-3759), 753(95%CI: 143-3594), and 2257(95%CI: 584-8731) for IV-WT*3-IV-WT, IV-WT*3-IV-O, IV-WT*3-PS-O, and IV-WT*3-RNA-O,respectively, corresponding to 3.4-, 2.7-, 3.6-, and 4.5-fold reductions of Omicron(Figure 1D). IV-O, PS-O, and RNA-O stimulated 2.7-, 4.7-, and 14.0-fold increases in the GMTs against BA.4/5, respectively, compared to IV-WT. Vaccination with a second booster dose of the Omicron-adapted vaccine increased the Nab titers against different Omicron sub-lineages.

We also measured the frequency of SARS-CoV-2-specific T cells using an ELISpot assay. The number of Omicron peptide-specific, interferon (IFN)-γ-producing T cells was significantly increased in individual mice vaccinated with the heterologous mRNA vaccine compared with those vaccinated with other vaccines (Figure 1E). These results support the use of the mRNA vaccine platform to induce an increase in T cell immune protection.

## Discussion

The threat posed by Omicron and its sub-variants is shown in the dramatically decreased Nab titers of COVID-19 vaccine-induced immune sera against Omicron compared to those for the prototype virus. To control the spread of Omicron, immunization strategies must prioritize boosting current vaccines that are ineffective against symptomatic Omicron infections [9]. We found that a homologous IV-WT booster rescued Nab titers against Omicron; however, the titers were markedly lower than those against the WT. These data are consistent with results obtained in humans vaccinated with three doses of BNT162b2 [10]. In contrast, as a secondary booster, the IV-O vaccine led to higher Nabs against Omicron, indicating that an Omicron-adapted booster vaccine is urgently needed. He et al. demonstrated that heterologous prime-boost may greatly increase immunogenicity [11]. In this study, PS-O and RNA-O vaccines were tested as heterologous boosters after three doses of IV-WT vaccination, and significantly enhanced Nab levels against Omicron and its sub-lineages compared to homologous boosting with the IV-O vaccine. These data are consistent with recent clinical results of Omicron-adapted vaccines produced by Pfizer [12], and support the prioritization of a heterologous boosting strategy.

There were several limitations to this study. First, all doses were administrated to mice. Our results must be confirmed in other animal models and humans. Second, we did not evaluate whether these four vaccination regimens protected against Omicron infection in human angiotensin-converting enzyme 2-translated mice. These limitations should be addressed in further studies.

In summary, immunization with three doses of IV-WT vaccines followed by an IV-O vaccine promoted increases in Nab levels against Omicron in mice. Heterologous booster doses of PS-O and RNA-O further elevated antibody levels against Omicron and its sub-lineages. An Omicron-adapted vaccine combined with heterologous boosting strategies may be effective for controlling COVID-19.

## Supplementary Material

Supplemental Material
