# Supplementary material for: Heterologous Omicron-adapted vaccine as a secondary booster promotes neutralizing antibodies against Omicron and its sub-lineages in mice
Source: Emerg Microbes Infect. 2022 Dec 12;12(1):e2143283. doi: 10.1080/22221751.2022.2143283 (PMC9754032; doi:10.1080/22221751.2022.2143283)
Supplement: Supplemental Material [file TEMI_A_2143283_SM6335.docx]

**Supplementary information**

**Materials and Methods**

**Mice, vaccines and vaccination**

For the first three injections of four-dose immunization, all mice (approximately 5–6 week old female BALB/c) were immunized intramuscularly with the IV-WT vaccine (1/5 of the human dosage, 0.1 mL) at days 0, 14, and 35. The mice were then divided into four groups and boosted with either the IV-O vaccine (n = 5, 1/5 of the human dosage, 0.1 mL), PS-O vaccine (n = 6, 1/5 of the human dosage, 0.1 mL), or RNA-O vaccine (n = 6, 1/5 of the human dosage, 6 μg) at day 56. Sera were collected at 14, 35, 56, and 70 days post-immunization to evaluate SARS-CoV-2-specific neutralizing antibody response as described below. All vaccines were donated by the manufacturers. IV-WT and IV-O vaccines are inactivated vaccines, which are created from African green monkey kidney

cells (Vero cells) that have been inoculated with SARS-CoV-2 virus (wild type and Omicron variant). The PS-O vaccine is a recombinant COVID-19 vaccine, using a chimeric multiple spike receptor-binding domain (RBD) of WT and Omicron as the antigen. RNA-O is an mRNA vaccine, which encodes the spike protein harboring the mutations found in Omicron. The sequences of Omicron spike protein in this study have been confirmed and contains the VOC-defining constellation of mutations.[1]

**Sera neutralizing antibody assay against SARS-CoV-2**

Animal immune sera samples were incubated at 56 ℃ for 30 min before use. Ppseudovirus neutralization assays were performed as previously described, and the results are expressed as the 50% neutralization titer [2]. A cytopathic effect-based live virus micro-neutralization assay was performed to determine the titers of the neutralizing antibodies against the WT, Delta, and Omicron strains. Briefly, 50 µL of plasma, starting from 1:8 in serial two-fold dilutions, was added to two wells of a cell culture plate. The plasma was mixed with 100 µL of titered virus and incubated at 37 ℃ in 5% CO_2_ for 2 h. Next, 1.0–2.0 × 10^5^ Vero cells were added to each well and incubated at 37 ℃ in 5% CO_2_ for 5 days to calculate the degree of the cytopathic effect compared to the values in the cell and virus controls. The 199 medium was supplemented with 6% new born calf serum, 1% penicillin, and 1% streptomycin. The neutralization antibody titers were calculated using the Reed-Muench method.

**ELISPOT Assay**

T cell immune responses in vaccinated mice were assessed using a mouse IFN-γ kit (BD Biosciences, cat: 551083) according to the manufacturer’s protocol. Briefly, plates coated with purified anti-mouse IFN-γ were blocked with RPMI 1640 containing 10% fetal bovine serum and incubated for 2 h. Immunized mouse splenocytes were plated at 2 × 10^5^ cells/well, with a peptide pool for the SARS-CoV-2 spike protein of the Omicron variant (1 mg/mL of each peptide), Concanavalin A as a positive control, or RPMI 1640 medium as a negative control. The peptide pool for the SARS-CoV-2 spike protein of the Omicron variant was synthesized by ChinaPeptides Co., Ltd. (Suzhou, Jiangsu Province, China). Each peptide contains 15 amino acids, and adjacent peptides overlap by nine amino acids. After incubation at 37 ℃ and 5% CO_2_ for 24 h, the plates were washed, and biotinylated anti-mouse IFN-γ antibody was added and incubated before 1 h incubation at room temperature. Following the addition of AEC substrate solution, the air-dried plates were evaluated using an automated ELISPOT reader CTL analyzer. The numbers of IFN-γ spot-forming cells per 1 × 10^6^ cells were calculated.

**Ethics statement**

All animal procedures were reviewed and approved by the Institutional Animal Care and Use Committee of the National Institutes for Food and Drug Control, China (Assurance No. 2020-B015).

**Reference**

1. World Health O. VOC profiles of Spike amino acid changes 2022 [cited 2022 September 14]. Available from: https://[www.who.int/docs/default-source/coronaviruse/s.pdf?sfvrsn=990a05c2_14](http://www.who.int/docs/default-source/coronaviruse/s.pdf?sfvrsn=990a05c2_14)
2. Nie J, Li Q, Wu J, et al. Establishment and validation of a pseudovirus neutralization assay for SARS-CoV-2. Emerging microbes & infections. 2020 2020/01/01;9(1):680-686.
